# Serial dependence in a simulated clinical visual search task

**DOI:** 10.1038/s41598-019-56315-z

**Published:** 2019-12-27

**Authors:** Mauro Manassi, Árni Kristjánsson, David Whitney

**Affiliations:** 1 Department of Psychology University of California, Berkeley, CA USA; 2 School of Psychology, University of Aberdeen, Kings College, Aberdeen, UK; 3 The Icelandic Vision Laboratory, School of Health Sciences, University of Iceland, Reykjavik, Iceland; 4 School of Psychology, National Research University Higher School of Economics, Moscow, Russian Federation; 5 Helen Wills Neuroscience Institute, University of California, Berkeley, CA USA; 6 Vision Science Group, University of California, Berkeley, CA USA

**Keywords:** Object vision, Pattern vision

## Abstract

In everyday life, we continuously search for and classify objects in the environment around us. This kind of visual search is extremely important when performed by radiologists in cancer image interpretation and officers in airport security screening. During these tasks, observers often examine large numbers of uncorrelated images (tumor x-rays, checkpoint x-rays, etc.) one after another. An underlying assumption of such tasks is that search and recognition are independent of our past experience. Here, we simulated a visual search task reminiscent of medical image search and found that shape classification performance was strongly impaired by recent visual experience, biasing classification errors 7% more towards the previous image content. This perceptual attraction exhibited the three main tuning characteristics of Continuity Fields: serial dependence extended over 12 seconds back in time (temporal tuning), it occurred only between similar tumor-like shapes (feature tuning), and only within a limited spatial region (spatial tuning). Taken together, these results demonstrate that serial dependence influences shape perception and occurs in visual search tasks. They also raise the possibility of a detrimental impact of serial dependence in clinical and practically relevant settings, such as medical image perception.

## Introduction

In everyday life, we continuously search for objects in the environment around us and classify them. We look for our wallet on a messy desk, for a friend in a crowd of people, or for a pair of socks in our room. Visual search can be literally life-saving in clinical and practically relevant settings. For example, in a medical screening setting, radiologists repeatedly search for signs of tumors in blurry radiological scan images, classifying them, judging their size, class, position and so on. Likewise, in airport security, transportation security officers need to identify prohibited items in carry-on luggage, in the midst of all possible kinds of personal belongings. The “human search engine” of radiologists and security officers is, like that of all humans, highly unreliable and fallible^1,2^; for example, more than 30–35% of mistakes in radiological screening are considered to reflect interpretation errors^3–5^.

An underlying assumption about visual search is that our current perceptual experience is independent of our previous perceptual experience. Recent results question this assumption, however. Our visual processing is characterized by serial dependencies, where what was seen previously influences (captures) what is seen and reported at this moment^6^. Serial dependencies occur for a variety of features and objects, including orientation^6,7^, position^8,9^, faces^10,11^, numerosity^12–14^ and other dimensions^15^. Such serial dependence is characterized by three types of selectivity. First, temporal tuning: serial dependence gradually decays over time^6,9,10,16^. Second, feature tuning: serial dependence occurs only between similar features, and not between dissimilar ones^6,9,17^. Third, spatial tuning: serial dependence occurs only within a limited spatial region and it is strongest when previous and current objects are presented at the same location^6,8,9,12,13^.

Based on these results, it was recently proposed that perception occurs through Continuity Fields (^6,10^, see also^18,19^): temporally and spatially tuned operators that bias our percepts towards previous ones through serial dependence. Continuity fields were proposed to be a beneficial mechanism that promotes perceptual stability in our ever-changing visual environment^6,7,10,20^. Over time, retinal images constantly change because our eyes, head and body move; because of many sources of external and internal noise; and because of discontinuities introduced by eye blinks. By recycling previously perceived features and objects, serial dependence helps us to represent a continuous and stable world despite noise and change.

In the autocorrelated world we live in, it is useful for our visual system to trade accuracy for increased perceptual stability. In artificially uncorrelated situations, however, serial dependencies can be detrimental for object recognition. The visual search tasks performed by radiologists in cancer image interpretation and officers in airport security screening are two pertinent examples. During these kinds of visual search tasks, observers often examine large numbers of uncorrelated images (tumor x-rays, checkpoint x-rays, etc.) one after another. We hypothesized that because of serial dependence, visual search performance on any given current x-ray image will be biased towards the previous x-ray, systematically altering image interpretation. To preview our results, we simulated a medical screening setting and found that simulated tumor shape classification was strongly impaired by an observer’s past visual experience. Classification errors increased and were pulled towards the previous x-ray content.

## Results

### Experiment 1

**Figure 1:**
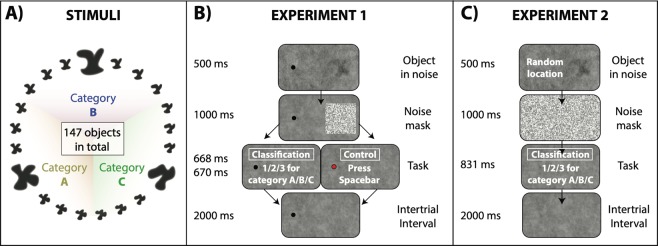
(**A**) We created three objects with random shapes (prototypes **A-B-C**, shown in a bigger size) and generated 48 morph objects in between each pair (147 objects in total). Each shape category was defined as the prototype **A/B/C** −/+24 morph units (49 morph units in total). (**B**) In Experiment 1, observers were asked to continuously fixate a black dot on the left part of the screen. On the right part of the screen, they were presented with an object hidden in noise, followed by a noise mask. Depending on the color of the fixation dot, they were then asked to classify the object as belonging to category **A–C** (black dot, 60% of the trials) or press the spacebar (red dot, 40% of the trials). After a 2000 ms inter-trial interval, the next trial started. (**C**) Experiment 2 was very similar to Experiment 1, except that there was no fixation dot (unconstrained free viewing), and the object was presented on each trial in a random location. On each trial, observers were asked to find the object on the screen (visual search task) and to classify the object (classification task) as belonging to category **A–C**.

In Experiment 1, we tested whether serial dependence influences recognition of visual stimuli in a classification task. In order to simulate the medical screening performed by radiologists, we created three objects with random shapes (simulated tumors A, B, and C) and generated 48 morph objects in between each pair (147 objects in total; Fig. 1A). On each trial, subjects viewed a random tumor-like object superimposed on a noisy background (simulated x-ray), followed by a mask of black and white pixels. Observers were asked to continuously fixate a black dot and the random object was presented 15° away from fixation in the right peripheral field (Fig. 1B). Observers were then asked to classify the object as belonging to category A, B or C (simulated tumor classification task; 66% of the trials). If the fixation dot turned red after mask appearance, observers were asked to simply press the spacebar (control task; 33% of trials).

**Figure 2:**
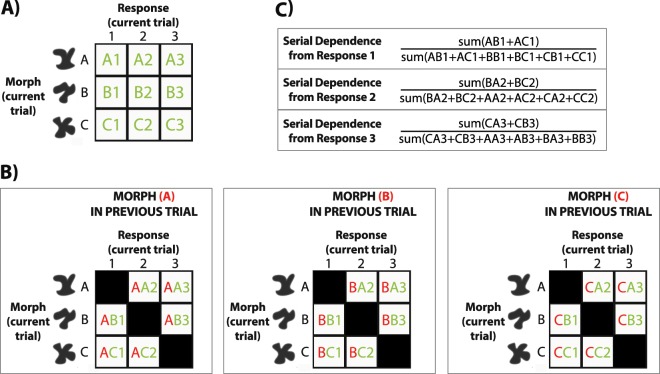
Temporal tuning analysis in Experiments 1 and 2. (**A**) We divided observers’ response frequency into a 3 × 3 matrix based on their responses 1/2/3 and the actual object category (**A/B/C**) on a current trial. (**B**) We then divided observers’ mistakes into three matrixes depending on whether the previous objects category was (**A/B/C**). First letter (in red) indicates morph category on the previous trial, second letter indicates morph category on the current trial (in green), and the number indicates classification response on a current trial (in green). Only mistakes were considered and hits were not taken into account (black squares on the diagonal line). (**C**) For a given response (1, for example), we summed mistakes when response and previous category were the same (**AB1,AC1**), and divided them by the sum of all the mistakes (**AB1,AC1,BB1,BC1,CB1,CC1**). This ratio yielded an index of serial dependence for a given responses (1 in this example). We then averaged the indexes across the three responses 1/2/3 and subtracted the chance baseline (33%).

**Figure 3:**
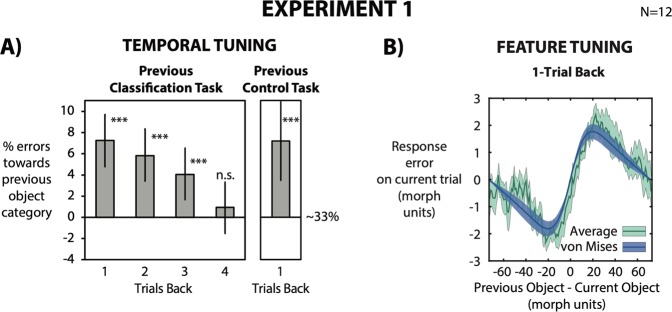
(**A**) We computed the percentage of errors towards the object category in the previous trial, normalized for the 33% chance level (chance baseline was confirmed by permuted null distribution). A 0% value indicates no bias from the previous object category, with a 33% chance of classifying the object as A/B/C. Higher and lower values than 0% indicate that a classification response was biased towards the previous object category (higher than 33%) or biased away (lower than 33%), respectively. For each observer we obtained a mean bootstrapped percentage by resampling the data with replacement 5000 times. Error bars are bootstrapped 95% confidence intervals, and p-value is based on the group bootstrapped distribution. Observers’ responses were strongly biased towards the previous object category up to 3 trials back (12 seconds back in time). Similar results were found when observers were asked to simply press the response bar in the 1-back trial, indicating that our results are not due to a response bias. (**B**) Response errors were computed as the distance between the response (corresponding to prototypes 1-A, 50-B or 99-C ) and current morph. Error plot as a function of the relative morph difference of the previous trial (1-back). The x-axis represents the difference between the previous and current object in morph units. The y-axis represents the error in the classification task (difference between response and object morph on current trial). The average error (green line) shows more negative response errors for a negative relative morph difference and more positive errors for a positive relative morph difference. Green shaded error bars indicate standard error across observers. The dark blue line indicates the average von Mises distribution across observers. Green shaded error bars indicate standard error across observers. Morph classification was attracted toward the morph seen on the previous trial. Importantly, it was tuned for similarity between previous and current morph (feature tuning).

Object classification accuracy, defined as correspondence between stimulus category (−/+24 morph units around the prototype shape A/B/C) and response, was 75.6 ± 6.3% (N = 12). As a categorical measure of serial dependence, we investigated whether errors in object classification were biased more towards the object category on the previous trial compared to other previous object categories. We computed the percentage of mistakes towards the shape category in n-back trials, and normalized the index by subtracting 33.33% (chance percentage level) from each percentage index (see Fig. 2 for an in-depth explanation). We then bootstrapped each subject’s data with 5000 iterations and reported the mean bootstrapped percentage as a metric of serial dependence (Fig. 3A).

Observers misclassified the object on a current trial as the shape in 1-back trials 7% more often than other shape categories. The misclassification lasted up to 3 trials back (1-back: p < 0.01; 2-back: p < 0.01; 3-back: p < 0.01; 4-back: p = 0.21; Fig. 3A, left panel). Average response time (RT) across subjects was 730 ± 200 ms; the 1-back object was presented on average ~4200 ms prior to the current trial object. The perceived object was therefore strongly attracted toward a random object seen more than 12 seconds prior, similar to the temporal tuning of serial dependence reported in the literature^6,9,11,16,17,21^.

In order to rule out a response bias, we analyzed the previous trials where observers gave an unrelated response (33% of the trials; control task) and the current trials where observers classified the object (66% of the trials; classification task). When observers were asked to withhold their classification responses in the previous trial, giving an unrelated “spacebar” response, serial dependence nevertheless occurred on the following trial (around 7%; Fig. 3A, right panel). In order to further control for unrelated biases and potential artifacts in our analysis that might manifest as spurious serial dependence^22^, we also computed serial dependence from future objects for 1, 2 and 3 trials forward. As expected, object classification responses were not significantly biased towards future objects (1-forward: 0.41%, p = 0.45; 2-forward: −0.39%, p = 0.52; 3-forward: 0.21%, p = 0.42). This confirms that neither systematic response biases, nor artifacts can explain the serial dependence effect.

Serial dependence did not simply occur at the response level (i.e., between responses 1/2/3), but was tuned for object similarity between the current and previous stimulus. We calculated perceptual error as the shortest distance along the morph wheel between the response categories (corresponding to 1, 50 and 99 morph units) and target objects (1–147 morph units). Each subject’s error on the current trial was compared to the difference in object morphs between the current and previous trial (Fig. 3B). We first computed the moving average of the response error as a function of the relative morph difference and averaged across observers (Fig. 3B, green line). In order to quantify feature tuning, we then fit a derivative-of-von Mises distribution to each subject’s running average data (Fig. 3B, blue line). When measuring the peak-to-trough amplitude and width of the derivative-of-von Mises distribution, response error was maximally biased with an average peak of 2.06 morph units for a relative morph difference of ±20 morph units, and gradually decreased with increasing morph difference (Fig. 3B; feature tuning).

Taken together, our results show that object classification is strongly biased towards previously presented objects up to 12 seconds in the past. In a task that mimics situations where observers assess target characteristics and classify them, such as in a medical screening setting, we show that serial dependence can have a *harmful* effect on object recognition, by biasing classification errors towards the previous x-ray content. As our visual system strongly expects constancy from one moment to the next, it tends to make our perception inaccurate in uncorrelated situations where extremely fine discriminations and accurate object recognition are required, such as during radiological screening or x-ray screening at airports.

### Experiment 2

It might be argued that in visual search, observers do not only have to identify and classify objects, but they must also scan the environment to find targets (for example, radiologists searching within x-ray images). For this reason, in Experiment 2 we randomized the position of the objects on each trial. Hence, observers were asked to first find the target^1^ and then classify it^2^. Our purpose was twofold. First, we aimed at mimicking the standard visual search tasks that radiologists typically perform on radiological scans; they are required to find tumors or tumor-like structures (if present) in several consecutive radiological scans, and to classify them (e.g., as malignant, benign, type, etc). Second, we investigated whether serial dependence in a visual search setting is affected by the spatial distance between current and previous objects, as shown by previous results^6,8,9,12,13^.

The procedure for Experiment 2 was identical to that of Experiment 1, except for the following changes. Observers’ fixation was unconstrained (free viewing). Object position was randomized on each trial within a 25° spatial window, and each object was further blurred (from 20 to 30 blur pixel radius). Observers were presented with an object in a random location, followed by a noise mask covering the entire screen. As in Experiment 1, they were asked on all trials to classify the object as belonging to category A/B/C (Fig. 1C).

Mean Accuracy was 65.7% ± 9.5% (N = 11). For each subject, we binned the trials into two groups based on the relative object location between current and previous trial: 0°–12.5° and 12.5°–25°. We then analyzed the influence of the previously presented object on a subsequent trial in these two groups of relative distances for 1–4 trials back. For a relative distance between 0° and 12.5°, serial dependence occurred for 1 trial back, biasing classification responses up to 5% (1-back: p < 0.01; 2-back: p = 0.18; 3-back: p = 0.24; 4-back: p = 0.16; group bootstrapped distribution), whereas for a relative distance between 12.5° and 25°, no serial dependence occurred (1-back: p = 0.22; 2-back: p = 0.45; 3-back: p = 0.17 4-back: p = 0.77; group bootstrapped distribution). Serial dependence was weaker compared to Experiment 1, with 5% of errors towards previous object category (compared to 7% in Experiment 1) and no effect for 2–3 trials back. This difference is strength may be due to the change in blur of the stimulus (see Method) or a shift from peripheral (Experiment 1) to foveal vision (Experiment 2), in accordance with evidence showing that serial dependence strongly depends on stimulus noise and attention^6,13,20^.

**Figure 4:**
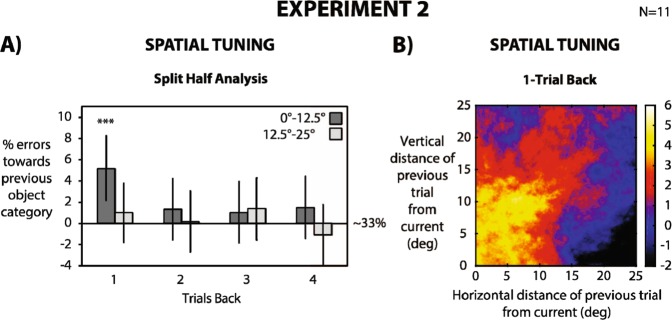
Stimuli and experimental procedures in Experiment 2 were the same as in Experiment 1, with the main difference that on each trial the tumor morph was presented at a random position. (**A**) We computed the percentage of errors towards the object category in the previous trial, normalized for the 33% chance level. Chance baseline was confirmed by a permuted null distribution. A 0% value indicates no bias from previous object category, with a 33% chance of classifying the object as (**A–C**). Higher and lower values than 0% indicate that classification response was biased towards the previous object category (higher than 33%) or biased away (lower than 33%), respectively. For each observer we obtained a mean bootstrapped percentage by resampling the data with replacement 5000 times. Error bars are bootstrapped 95% confidence intervals, and p-value is based on group bootstrapped distribution. For each subject, trials were divided into two groups based on the relative spatial distance between current and n-back trials (0–12.5° and 12.5°–25°). Observers’ responses were biased towards the previous object category up to 1 trial back, only when presented within a spatial window within 0 and 12.5° (dark bars). (**B**) This percentage of responses was computed between morph tumors at different locations within a two-dimensional rolling window over the relative positions of the previous and current stimuli. Color-coding shows the amplitude (in percentage) of biased responses computed at each window location. Observers’ responses were maximally biased toward previous stimuli in a relative spatial range of 10–12°.

In order to further characterize the spatial tuning of the effect, we computed serial dependence between morph tumors at different locations within a two-dimensional rolling window, over the relative positions of the previous and current stimuli (Fig. 4B; see Methods section): serial dependence gradually decreased with increasing relative spatial distance between the current and previous object. Taken together, our results show that serial dependence also occurs in a visual search setting reminiscent of a radiologist’s (visual search and classification), and that serial dependence strongly depends on the relative location of the target relative to the preceding ones.

## Discussion

Visual search in a simulated medical screening paradigm is serially dependent; misclassifications were biased towards the object category in the previous trial by up to 7%. Serial dependence could therefore be a substantial source of error in several critical visual search situations, like for example cancer image interpretation^1^ and screening at airport checkpoints^23^. Importantly, our results show that this source of errors is not unavoidable. Serial dependence did not indiscriminately occur across time, features, and space. It lasted up to 12 seconds (Figs. 3A and 4A), occurred only between similar objects (Fig. 4B) and within a spatial window of 10–12.5° (Fig. 4B).

Our results are not due to a mere response bias for three main reasons. First, serial dependence on a current trial occurred also when, in the previous trial, observers were asked to give a “spacebar” response completely unrelated to the stimulus (Control Task, Fig. 1B). Hence, a previous classification response (or a perceptual decision) per se is not necessary for serial dependence to occur. Second, serial dependence did not simply occur between responses (sequence 1/2/3) but it was specifically tuned to stimulus characteristics. It impacted classification performance depending on object similarity on the current and previous trials, with a peak for an object morph difference of 20 morph units (Fig. 3B; feature tuning). Third, a response bias would indiscriminately occur across the entire visual field, but we showed in Experiment 2 that serial dependence is spatially tuned (Fig. 4) in accordance with previous results^6,8,9,12,13^.

Previous studies have investigated the impact of previous visual experience on visual search, but they mostly involved reaction time and eye movements measurements^24,25^. For example, search speed for a target is faster if previous and current targets share the same features^26–33^ or location^27,34–38^. Between-trial repetition of features and locations also improves recognition of briefly presented masked items^39–41^. Additionally, Chetverikov, Campana et Kristjánsson^42,43^ showed that observers can learn the shapes of distributions of items to be ignored if the distractors come from the same distribution on consecutive trials. Interestingly, eye movements are substantially influenced by memory for past fixation locations, although this was found in a detection task paradigm^25^.

Previous visual experience can determine image recognition also in the medical screening domain. Kompaniez *et al*.^44^ adapted normal observers for 60 seconds to image samples of dense or fatty tissue, and they asked them to judge the appearance of intermediate images in a texture matching task. Long exposure to dense images caused an intermediate image to appear more fatty (and vice versa), thus leading to a negative aftereffect. In a similar paradigm, adaptation to radiological images was also found to modulate RT^45^. Whereas these two results show that long adaptation to previous history (60 seconds) can affect recognition and discrimination *away* from the past, our current results show for the first time that a much shorter exposure (500 ms) can strongly bias object recognition and discrimination in a visual search task *towards* the past. Future research will have to determine under which specific conditions these two opposing biases in visual perception determine recognition performance.

Our results are in accordance with the idea of Continuity Fields, spatiotemporally tuned operators where similar features and objects are integrated over time^6,7,10^. Our serial dependence effects exhibited the three main defining criteria of Continuity Fields: temporal tuning, feature tuning, and spatial tuning. The main purpose of Continuity Fields is to favor object stability by merging similar information over time (see also 18, 19); they are therefore a beneficial mechanism in the autocorrelated world in which we live. Here, we show another side of the same coin: in non-correlated situations, such as visual search for tumors in radiological scans, Continuity Fields can be detrimental, impairing classification performance toward previous x-ray content.

Our results cannot clearly disentangle whether serial dependence occurs on a perceptual^6,9,12,20,46,47^ or decisional/memory level^8,17,48^. According to the first account, serial dependence changes stimulus appearance, biasing the current stimulus towards the previous one^6,12,20^, whereas under the second account serial dependence biases our internal decision or memory towards the previous one^8,17,49^. Nevertheless, our, results lean toward the perceptual hypothesis, because serial dependence is selective to the characteristics of the stimulus (featural tuning) and its location in the visual field (spatial tuning). In addition, even when observers do not withhold decisions on the previous trial (as in the control task condition; Fig. 3A), serial dependence still biased the next stimulus (as opposed to Pascucci *et al*.^50^). However, it must be mentioned that these accounts are not necessarily mutually exclusive. Different mechanisms may interact with one another on several levels^49^; decision and memory may change target appearance and the other way around. More importantly, independent of the underlying mechanism(s) of serial dependence, our results carry a much broader and more pragmatic message: that a considerable percentage of errors in medical visual search may be due to sequential effects from previous perceptual experiences.

Previous research has shown that visual search in a clinical setting is determined by various factors. Attentional guidance plays a crucial role: by focusing their attention on spotting tumors in x-ray images, radiologists were shown to miss objects as salient as gorillas^51–54^. Different search strategies can be more effective than others: drilling through 3D images in depth (drillers strategy) can be more accurate than scanning each image before moving to the next one (scanners strategy, 55). Target prevalence and distribution also play a crucial role in visual search; target misses strongly increase with decreasing target prevalence^56–61^.

Serial dependence may play an important role in all these factors. Serial dependence depends on attention^6,12^, and should particularly impair the tumor shapes radiologists are looking for, independently of the background image content. Regarding search strategy, drilling through 3D images should keep the image more autocorrelated across time, and hence the Continuity Field should have a beneficial impact in stabilizing our percept. Conversely, scanning random locations in each image before going to the next one will lead to potentially less autocorrelated information and the Continuity Field should have a detrimental effect, biasing perception towards previous parts of the same radiological scan. Consistent with this, radiologists make more mistakes with the scanning than the drilling strategy^55^. Regarding target prevalence, in our experiments each object category was equally likely to be presented on the screen (33%), but under certain visual search situations targets are extremely rare. For example, malignant tumors for radiologists or threats in carry-on luggage for transportation security officers are rarely encountered. When searching through several consecutive empty scans, serial dependencies should continuously bias our percept towards more frequent objects, making it even more difficult to recognize rare targets for what they actually are.

One concern could be that our stimuli were not realistic. Indeed, our stimuli and task were intentionally controlled for the sake of measuring potential sequential dependencies. Our stimulus and task, however, are reminiscent of those that radiologists routinely encounter. Radiologists are usually asked to look at radiological images^1^, search for tumors^2^, and classify them as malignant or benign depending on their shape and location^3^. Accordingly, we adapted standard serial dependence paradigms to mimic a noisy background^1^, a target similar to a tumor shape^2^ and a classification task^3^. Hence, we consider our results as “proof of concept” that serial dependence can detrimentally affect clinically and practically relevant visual search settings. Of course, whether any particular visual search task (e.g., mammography, cytology, etc) or individual subject group (radiologist, resident, ultrasound technician) suffers from serial dependence remains an open and important question for further investigation.

Future research is needed to show effects of serial dependence in actual radiological screening settings. First, having context-related stimuli is crucial. Future research will present sequences of actual x-ray with actual tumors, instead of simulated ones. Second, in our experiments the simulated tumor was presented for a relatively short duration (500 ms). Radiologists may fixate radiological scans for a much longer period before they make a decision. However, there is psychophysical evidence that radiologists are capable of extracting valuable information from an image at first glance, without lengthy examination of it^62–67^. Tumor recognition is well above chance with short duration stimuli, but it increases to nearly perfect with unlimited viewing. Accordingly, future research will test whether sequential effects also occur with longer x-ray presentations. Of course, as mentioned above, long exposure may lead to negative perceptual aftereffects, leading to opposing results^44,45^. Third, there are well-known target frequency effects on visual search^59,61,68–72^. In our experiments, each shape category was equally likely to appear on any trial (33%), whereas in radiological screening the chance of encountering a radiological scan with a tumor is very low, around 1–2%. Future research will test whether having a less frequent target will increase or decrease serial dependence. On one hand, having a rare target may lead to the well-known phenomenon of satisfaction of search, i.e. failure to detect subsequent abnormalities after identifying an initial one^73–75^. This phenomenon would work against serial dependence, thus leading to a “no tumor” response when the previous x-ray contained one. On the other hand, such a low tumor frequency means also higher exposure to non-tumor stimuli (e.g., benign masses, cysts, etc.), and may therefore boost serial dependence strength. That is, benign tumors and non-tumor structures could generate serial dependence as well.

In addition to the potential importance for clinically and practically relevant settings, our results also provide new insights into the mechanism(s) underlying serial dependence. They show that serial dependence can bias perceptual decisions for simple stimuli like shapes, thus making dissimilar shapes appear more similar than they actually are. This shows that serial dependence can occur with low-level^6,76,77^ and high-level stimuli^10,13,78^. In the recent literature, serial dependence has been assessed with three main task types: stimulus adjustment and matching^6,10,17^, two-alternative forced choice tasks^6,12,17^, as well as rating scales and magnitude estimation^15,79,80^. Here, we show that serial dependence can also be investigated through a three-alternative forced-choice classification task, which has the advantage of not presenting any visual stimulus during the response. Finally, our results highlight the importance of serial dependence in the domain of visual search^81–83^. They show that serial dependence can strongly bias subsequent search for items, but only if the items are similar and are presented within a limited temporal and spatial window.

## Conclusion

Our results show 1) that visual search is serially dependent, and 2) that this occurs in a simulated clinically relevant setting; past experience can affect recognition, biasing classification errors towards previous simulated x-ray content. Serial dependence impairs classification performance within defined temporal, featural, and spatial boundaries in line with the defining characteristics of Continuity Fields. Importantly, the limits of these three types of tuning open the door to potential strategies which may mitigate their detrimental effects.

## Method

All experimental procedures were approved by and conducted in accordance with the guidelines and regulations of the UC Berkeley Institutional Review Board. Participants were affiliates of UC Berkeley and provided informed consent in accordance with the IRB guidelines of the University of California at Berkeley. All participants had normal or corrected-to-normal vision, and were all naïve to the purpose of the experiment. Twelve subjects (6 females; age = 20–31 years) participated in Experiment 1. Eleven subjects (7 females; age = 19–28 years) participated in Experiment 2. Stimuli were generated on a Macintosh computer running Matlab PsychToolbox^84^ and presented on a gamma-corrected CRT Sony Multiscan G500 monitor. The refresh rate of the display was 100 Hz and the resolution 1024 × 768 pixels. Stimuli were viewed from a distance of 57 cm. Subjects used a keyboard for all responses (“1–3” keys for the classification task, and spacebar for the control task).

### Stimuli

The stimuli consisted of dark-gray shapes based on 3 original prototype shapes (A/B/C; Fig. 1A). A set of 48 shape morphs was created between these prototypes, resulting in a morph continuum of 147 objects. Approximate width and height were 4°. Each object was blurred in Photoshop by using a gaussian blur filter. Blur pixel radius was 20 in Experiment 1 and 30 in Experiment 2. On each trial, a random object was inserted on a random brownian noise background (1/f^2^ spatial noise) with a 50% transparency level. The object embedded in the noisy background was presented for 500 ms, followed by a 1000 ms noise square mask (6° size) of black and white pixels (to reduce afterimages). Subjects were then asked to classify the object as belonging to category A/B/C by pressing 1/2/3 on the keyboard (classification task). After a 2000 ms delay, the next trial started. In a preliminary session, observers completed a practice block of 108 trials (Experiment 1) and 54 trials (Experiment 2), where the three prototype stimuli were shown on the screen when observers were asked to make a response. In addition, observers were continuously familiarized with the three prototype stimuli by seeing them on the screen for 10 seconds at the beginning of each block.

### Experiment 1

Observers were asked to continuously fixate a black dot (0.2° radius) and on each trial the objects were presented at 15° eccentricity in the right peripheral field. On 66% of the trials, the fixation dot was black and observers were asked to perform the classification task (Classification task). On 33% of the trials, the fixation dot turned red after the mask stimulus and observers were asked to simply press the spacebar (Control task). Observers performed 20 blocks of 54 trials each (Fig. 1B). Mean reaction time was 668 ± 15 ms in the Classification Task and 670 ± 9 ms in the Control Task.

### Experiment 2

There were no fixation constraints and the location of the object was changed randomly from trial to trial within a spatial square window of 25°. On each trial, observers were asked to find the object within the noisy background and classify whether it belonged to category A, B, or C. Observers performed 10 blocks of 108 trials each (Fig. 1C). Mean reaction time was 831 ± 346 ms.

### Temporal tuning

As a measure of performance on the classification task, we computed the accuracy for each observer, defined as the correspondence between response 1/2/3 and morph category A/B/C. Trials were considered lapses and excluded if response times were longer than 5 seconds. On average, less than 5% of data was excluded. Observers were removed from the experiment if their overall classification accuracy was lower than 50% or higher than 85% (two observers removed). Even including these two observers in the analysis did not significantly change the overall pattern of results. If observers classified all objects more than 60% of the times with a specific category for the first 2 blocks, thus showing a strong bias towards a specific object or response, the experiment was immediately terminated (two observers removed).

As a measure of serial dependence, we computed the percentage of errors towards the previous object category on an n-back trial (Fig. 3A). First, we computed the number of erroneous responses A/B/C given object categories A/B/C on an n-back trial. Second, for each response 1/2/3 we summed up the number of erroneous responses where previous object category and current response were the same, and divided it by the sum of erroneous responses for all previous object categories. The ratio was computed for each response 1/2/3. For example, the number of erroneous responses A when the previous Object Category was A was divided by the number of erroneous responses A when the previous Object Category was A + B + C. Third, we averaged the three obtained percentages across the three response categories 1–3 and subtracted the chance baseline 33.3% to normalize our index (Fig. 2).

The final index denotes by how much the categorical responses on a current trial are biased by the previous stimulus category. Positive values indicate a positive serial dependence, negative values indicate a repulsion, and zero indicates no bias. For each subject’s data, we generated confidence intervals by calculating a bootstrapped distribution of the model-fitting parameter values by resampling the data with replacement 5000 times. On each iteration, we recalculated the error percentage to obtain a bootstrapped percentage index for each subject. P-values were calculated by computing the proportion of percentages in each subject’s distribution (standard index – bootstrapped distribution) that were greater than or equal to zero. In order to empirically confirm the 33.3% chancel level, we also generated a null distribution of percentage errors for each subject using a permutation analysis. We randomly shuffled the order of the previous n-back trial and recalculated the error percentage for each iteration of the shuffled data. We ran this procedure for 5000 iterations. The average of the null distribution across observers was 33.32% (s.d. 0.07) in Experiment 1 and 33.33% (s.d. 0.04) in Experiment 2.

### Feature tuning

Relative morph difference (x-axis) was computed as the difference in morph units between the previous and current object (previous object morph–current object morph). Response error (y-axis) was computed as the difference between the response in the classification task, corresponding to morph 1 (prototype A), 50 (prototype B) and 99 (prototype C), and the current morph (current response – current object morph). For each observer, we computed the running circular average within a 15 morph units window and averaged the moving averages across all observers (Fig. 3B). We quantified feature tuning by fitting a von Mises distribution to each subject’s running average data. Peak-to-trough amplitude of the von Mises distribution across observers was significantly higher than zero (average across observers 2.06 morph units, s.d. 0.97; t^11^ = 7.34, p < 0.01) for an average width of ±19 morph units.

### Spatial tuning

In order to measure the spatial tuning of serial dependence, we binned trials according to the distance between the current and previous object locations (Fig. 4A). Distance between successive object locations was computed as:

$$\sqrt{{(xcurrent-xprevious)}^{2}+{(ycurrent-yprevious)}^{2}.}$$

We divided trials into two main groups: 0°–12.5° (Fig. 4A; dark bars), and 12.5°–25° (Fig. 4A; bright bars) for n-back trials. For each subject, we extracted the first 400 trials in both groups and computed the bootstrapped percentage of serial dependence within each group (5000 iterations; see Temporal tuning section).

We repeated the analysis using the two-dimensional spatial separation between successive trials (i.e., considering x distance and y distance separately; Fig. 4B). The rolling window was a circle with a starting radius of 8° that parametrically increased with increasing diagonal distance until 16° in order to collect a similar amount of data at each point. From each subject, we extracted the first 800 trials and collapsed all the data into a “super subject” (9600 trials in total). We then computed the bootstrapped percentage of serial dependence (1000 iterations) at each spatial separation between the current and previous trial. In Fig. 4B, a value of 0 on both axes (relative vertical and relative horizontal distance) indicates that the objects were presented at the same location on the current and the previous trial. A value of 25° on both axes indicates that the objects were presented at 25°of horizontal and vertical distance on both the current and previous trial (35° of diagonal distance). In order to increase the number of available trials, we collapsed left-right and up-down quadrants into a unique quadrant.

## Data Availability

All relevant data are available from the authors.
