# Author Correction: Serial dependence in a simulated clinical visual search task

**DOI:** 10.1038/s41598-020-60115-1

**Published:** 2020-02-18

**Authors:** Mauro Manassi, Árni Kristjánsson, David Whitney

**Affiliations:** 1 Department of Psychology University of California, Berkeley, CA USA; 2 School of Psychology, University of Aberdeen, Kings College, Aberdeen, UK; 3 The Icelandic Vision Laboratory, School of Health Sciences, University of Iceland, Reykjavik, Iceland; 4 School of Psychology, National Research University Higher School of Economics, Moscow, Russian Federation; 5 Helen Wills Neuroscience Institute, University of California, Berkeley, CA USA; 6 Vision Science Group, University of California, Berkeley, CA USA

Correction to: *Scientific Reports* 10.1038/s41598-019-56315-z, published online 27 December 2019

The Acknowledgements section in this Article is incomplete.

“We would like to thank Allison Yamanashi, Yuki Murai, and Zhimin Chen for helpful comments on data analysis and preliminary drafts of the manuscript. This work was supported in part by the Swiss National Science Foundation fellowship P2ELP3_158876 (M.M.).”

should read:

“We would like to thank Allison Yamanashi, Yuki Murai, and Zhimin Chen for helpful comments on data analysis and preliminary drafts of the manuscript. This work was supported by the Swiss National Science Foundation fellowship P2ELP3_158876 (M.M.) and the National Institutes of Health grant R01 CA236793.”

